# Evaluation of 'see-see and treat' strategy and role of HIV on cervical cancer prevention in Uganda

**DOI:** 10.1186/1742-4755-7-4

**Published:** 2010-05-10

**Authors:** Twaha Mutyaba, Florence Mirembe, Sven Sandin, Elisabete Weiderpass

**Affiliations:** 1 Department of Obstetrics and Gynecology, Makerere University, Medical School, P.O. Box 7072, Kampala, Uganda; 2 Department of Medical Epidemiology and Biostatistics, Karolinska Institutet, Stockholm, Sweden; 3 Department of Etiological Research, Cancer Registry of Norway, Oslo, Norway; 4 Department of Genetic Epidemiology, Samfundet Folkhalsan, Helsinki, Finland; 5 Department of Community Medicine, University of Tromsø, Tromsø, Norway

## Abstract

**Background:**

There is scant information on whether Human Immunodeficiency Virus (HIV) seropositivity has an influence on the outcome of treatment of precancerous cervical lesions using cryotherapy. We studied the prevalence of cervical abnormalities detectable by visual inspection and cervical lesions diagnosed by colposcopy according to HIV serostatus and described the outcomes of cryotherapy treatment.

**Methods:**

Trained nurses examined women not previously screened for cervical cancer using visual inspection with acetic acid (VIA) and Lugol's iodine (VILI) in two family planning/post natal clinics in Kampala, Uganda, from February 2007 to August 2008. Women with abnormal visual inspection findings were referred for colposcopic evaluation and HIV testing. Women with precancerous cervical lesions detected at colposcopy were treated mainly by cryotherapy, and were evaluated for treatment outcome after 3 months by a second colposcopy.

**Results:**

Of the 5 105 women screened, 834 presented a positive screening test and were referred for colposcopy. Of these 625 (75%) returned for the colposcopic evaluation and were tested for HIV. For the 608 (97.5%) women in the age range 20-60 years, colposcopy revealed 169 women with cervical lesions: 128 had inflammation, 19 had low grade squamous intraepithelial lesion (LGSIL), 13 had high grade squamous intraepithelial lesion (HGSIL), 9 had invasive cervical cancer and 2 had inconclusive findings. Detection rates per 1 000 women screened were higher among the older women (41-60 years) compared to women aged 20-40 years. They were accordingly 55% and 20% for inflammation, 10% and 2% for LGSIL, 5% and 2% for HGSIL, 6% and 1% for invasive cervical cancer.

Of the 608 women, 103 (16%) were HIV positive. HIV positivity was associated with higher likelihood of inflammation (RR = 1.7; 95% CI: 1.2-2.4).

**Conclusions:**

Detection rates were higher among older women 41-60 years. Visual inspection of the cervix uteri with acetic acid (VIA) and Lugol's iodine (VILI) used as a sole method for cervical cancer screening would entail significant false positive results. HIV seropositivity was associated with a higher prevalence of inflammatory cervical lesions. In view of the small numbers and the relatively short follow up time of 3 months, we could not make an emphatic conclusion about the effect of HIV serostatus on cryotherapy treatment outcome.

## Background

Cervical cancer is the second most common cancer among females worldwide, accounting for 452 000 new patients per year. It is the most common female cancer in sub-Saharan Africa alone and 57 000 estimated new cases of cervical cancer occurred in the year 2000, comprising 22% of all cancers [1]. In Uganda, cervical cancer is the most common female cancer with an estimated age standardized incidence rate of 40.7 per 100 000 [2].

The incidence of squamous cell carcinoma of the cervix has declined in the high income countries over time largely due to effective screening services using the Pap smear [3-5]. Such programs which use cytological screening are too expensive both materially and at the organizational level for most low income countries [6,7]. A situational analysis of cervical cancer screening services in 5 countries in eastern, central and southern Africa including Uganda, found insufficient cervical cancer screening services at most health units [8]. In a few countries where they exist, scant information on evaluation of the programs is available.

Persistent cervical infection with high risk human papillomavirus (HPV) has been established as the necessary cause of cervical cancer [9,10].

Several studies have demonstrated an increased risk of invasive cervical cancer among HIV positive women. Also the US Centers for Disease Control and Prevention included cervical cancer as one of the AIDS defining illnesses [11]. However some studies, in particular in sub-Saharan Africa, have found no association between HIV seroprevalence and invasive cervical cancer [12-15]. Competing risk of mortality from other conditions associated with HIV has been suggested as a possible explanation for this lack of consistency [14]. Similar sexual behavioural factors predispose individuals in acquiring of both HPV and HIV. The immunosuppressive effects of HIV has been reported to contribute to multiple HPV infections [16], persistence of high risk HPV infections [17], and the progression of HPV induced neoplastic cervical changes [18-22].

Uganda has succeeded in reducing HIV prevalence substantially in the last decade from 15-30% in 1992 [23] to currently 5-8% in the general population, with uneven distribution among men (5%) and women (8%) aged 15-49 years [24].

Guidelines on cervical cancer screening among HIV positive women differ from country to country. For newly diagnosed HIV positive women most developed countries recommend two screening tests with a six month interval, followed by annual screening [25,26]. The feasibility of such a strategy is doubtful for poor countries in particular because of difficulties in follow-up of patients. Recent evidence indicates that immunosuppression, as evidenced by reduced CD4 counts - and not HIV positivity per se - is responsible for the HPV persistent infection, recurrence and rapid progression [27,28]. The increasing availability of antiretroviral treatments will have implications for the screening and treatment regimens for cervical precancerous lesions among HIV seropositive women, but these are so far undocumented in sub-Saharan Africa. Information is required to develop feasible guidelines for poor countries, such as Uganda, where the prevalence of both HIV and cervical cancer is high.

Despite the great promise of the newly developed HPV vaccines, availability of HPV vaccines will not negate the need for screening [29]. Since cytology based screening is not feasible in low resource countries like Uganda, alternative screening methods have been suggested. A recent study from India showed significant reduction from cervical cancer deaths by single HPV testing followed by treatment [30]. Though superior, HPV DNA screening is still relatively expensive and in the poorer countries less costly screening methods such as visual inspection with acetic acid (VIA) in 'see and treat' strategies may remain the only available and feasible method for the near future. Visual inspection has been found to be safe and acceptable when performed by nurses [31] although its impact in preventing advanced cervical lesions is still controversial with specificity consistently reported as lower than cytology. This is a situation that may lead to over diagnosis and over treatment [32].

We present results from a see-see and treat cervical screening program in Uganda, describing the prevalence of cervical lesions as diagnosed by colposcopy, detection rates by age group and the outcome of the cervical lesion treatment using cryotherapy.

## Methods

### Setting and screening routine

The study was carried out in Mulago, the national referral and teaching hospital in Uganda, a country where so far only opportunistic cervical cancer screening is offered in very few centres mainly using visual inspection. Two family planning/postnatal clinics that are run by nurses/midwives were the primary health care facilities. The 9 nurses participating in this study had experience on use of the vaginal speculum since they used it routinely for inserting intrauterine contraceptive devices; they were intensively trained for a period of one month by a gynaecologist on screening using visual inspection with acetic acid and Lugol's iodine. Special emphasis was made on recognition of the squamo-columnar junction, the transformation zone, acetowhitening and colour changes resulting from application of Lugol's iodine. A gynaecologist was always available in the clinics throughout the study period for consultation whenever the nurses were uncertain about their findings. At the beginning of each day when the clients were assembled, group health education sessions were held about the available reproductive health services. The education included relevant information about the extent of the problem of cervical cancer, causes and risk factors, symptoms of the disease and treatment options. Lastly and in detail, they were educated on the prevention of the cancer including the details of the available screening procedure. At the end those who were eligible for screening were offered the screening test. Women, who were not currently menstruating, were not pregnant, who had never been screened for cervical cancer before and had no history of hysterectomy were eligible for screening in the study. Eligible women were then informed of the study in detail and a signed informed consent was obtained from those who agreed to participate in the study. A structured interview using a questionnaire was performed by the nurses, including information on education, socioeconomic status, reproductive and medical history. The visual screening was performed by application of freshly prepared 5% acetic acid to the cervix. After one minute, the nurse inspected the cervix for acetowhitening in the transformation zone close to the squamocolumnar junction. If there was no acetowhitening, it would be regarded as a negative test. If there was acetowhitening, the nurse would then apply Lugol's iodine and observe the colour changes. A normal cervix would turn mahogany brown. Non uptake or mustard yellow colouring would imply an abnormality. The nurse would inform the woman about the visual inspection findings, and those with a positive screening test would be referred for colposcopy after a week. This to allow a few days (3-5) for Lugol's iodine to disappear from the cervix, which would otherwise obscure the vision of cervical vasculature and the acetowhitening which is essential in colposcopic evaluation. The work of the nurses was supervised and monitored by a trained gynaecologist.

### HIV testing

All women who returned for the colposcopic examination were tested for HIV. We used the Abbott Determine HIV-1/2 qualitative immunochromatographic test manufactured by Abbot Japan co., ltd, Minato-Ku, Tokyo, Japan. Test results were validated using 2 other tests: The ChemBio HIV 1/2 Sta-Pak Dipstick, manufactured by ChemBio Diagostics Systems, Inc., 3661 Horseblock Road, Medford, NY 11763, USA, and The Trinity Biotech Uni-Gold HIV Test manufactured By Trinity Biotech Plc, Bray Co Wicklow, Ireland. The women had been informed about the HIV testing prior to their accepting to participate in the study. Post test counselling was done and where necessary women were directed to the relevant clinics that offer HIV care services.

### Colposcopic evaluation and treatment of lesions

Blind to the HIV status of the woman, colposcopy was done by the same investigator who graded the lesions using the Reids colposcopic index [33]. The index considers 4 colposcopic signs; lesion margin, colour of acetowhitening, blood vessels and iodine staining. It permits objective differentiation between squamous intraepithelial neoplasm (SIL) as low-grade cervical lesions (LGSIL) and high-grade lesions (HGSIL). Use of the index helps direct the clinician to perform a biopsy of the most significant abnormal cervical lesions. Findings were recorded on a scale of increasing severity as: normal, inflammation, atypia/CIN/condylomata/wart/leukoplakia/HPV change, CIN2-3, invasive carcinoma or inconclusive.

After taking a colposcopic guided biopsy, precancerous lesions were treated immediately using cryotherapy or LEEP if the lesion extended over more than 75% of the transformation zone. The cryotherapy used nitrous oxide gas. All women who were treated were given a course of antibiotics, (Metronidazole and Doxycycline) for a week.

We did not take biopsies from the cervix that appeared normal on colposcopy or where the colposcopist was convinced the diagnosis was inflammation. Punch biopsy has its own complications and we did not want to expose the women to any added morbidity. The women were told to come back for re-evaluation after 3 months. However, they could come back earlier if they experienced complications like bleeding or persistent foul smelling vaginal discharge. Those who had other cervical conditions like infections were appropriately treated and those with cervical polyps or obvious cancer were admitted to the gynaecological ward for appropriate management.

### Follow up evaluation

The women who had precancerous lesions and were treated had a colposcopic re-evaluation after 3 months by the same colposcopist to assess the outcome of treatment i.e. persistent lesions, infection/inflammation, and cervical stenosis. The information was duly recorded on their data forms and women informed of the findings.

### Data analysis

We described the demographic characteristics of the women who were screened. We described the VIA/VILI test results and compliance with referral for colposcopy. We report on the HIV serostatus of the women who returned for colposcopy. We computed the detection rates by age group per 1 000 women screened, basing it on the colposcopy diagnosis. We did not use the histology results in the analysis as we had not taken biopsies where colposcopy diagnosis was normal or an inflammation, due to ethical reasons. The computation of detection rates was done for women between 20-60 years of age since no lesions were found among women outside this age bracket, since they were only 2.5% of the total. The software Stata version 10 was used for all the data analyses.

### Ethical considerations

The study was cleared by the institutional review board of Makerere Medical School and the National Council for Science and Technology. During the study period, the screening service was offered to all eligible women, even those who declined to participate in the study. Those who participated in the study gave a written informed consent. All women were clearly informed that if they did not desire to participate in the study they would receive the same care, and if participating that they could withdraw their participation at any time; and that their eventual withdrawal would not affect treatment. All screening and treatment was free of any cost to the patient except the transportation costs of returning for the colposcopy visit, which they themselves had to meet.

## Results

**Figure 1:**
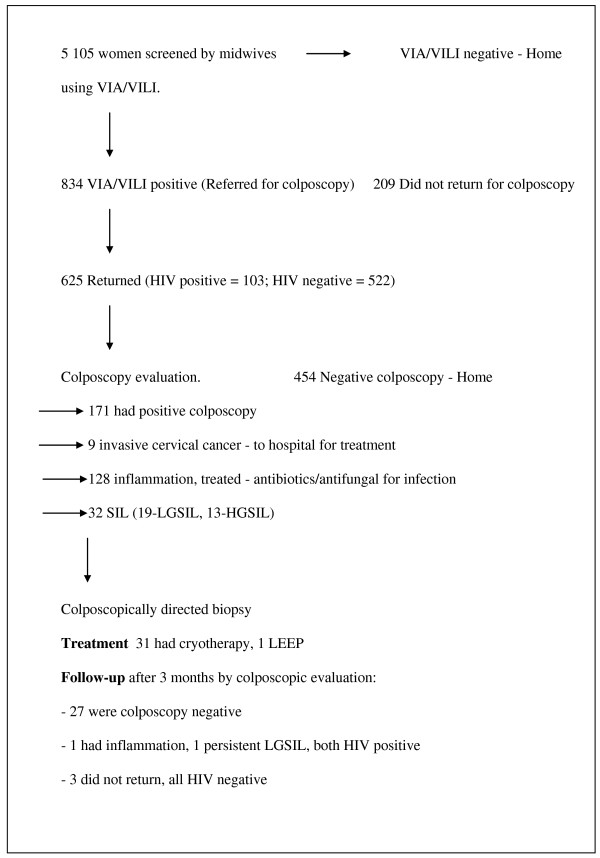
**Study flow chart**.

Altogether 5 105 women were screened between February 2007 and August 2008. Follow up was up to 30^th ^November 2008. The flow of women into the study is summarized in Figure 1. The demographic characteristics of the women screened are summarised in Table 1. The majority of women screened (97.5%) were between 20-60 years of age. 70% had started sex before 20 years of age, 16% had had more than 5 pregnancies. 95% had achieved at least primary school education. Only 24% could be considered high income earners.

**Table 1:** Demographic characteristics of the women screened

	Number	%
**Age groups (years)**		
< 20	92	1.8
20-40	4 119	80.7
41-60	860	16.9
> 60	34	0.7

**Total**	**5 105**	**100**

**Education**		
Inst/University	1 613	31.6
Secondary	1 869	36.6
Primary	1 385	27.1
Nil/Unknown	238	4.7

**Total**	**5 105**	**100**

**Income status**		
High	1 198	23.5
Medium	1 460	28.6
Low	2 447	47.9

**Total**	**5 105**	**100**

**Residence**		
Urban	4 475	87.7
Rural	630	12.3

**Total**	**5 105**	**100**

**Onset of sex**		
< 20 years of age	3 608	70.7
≥ 20 years of age	1 497	29.3

**Total**	**5 105**	**100**

**Total pregnancies**		
> 5	795	15.6
≤ 5	4 310	84.4

**Total**	**5 105**	**100**

**Distance from Mulago**		
≤ 10 km	4 112	80.5
> 10 km	993	19.5

**Total**	**5 105**	**100**

A positive visual inspection screening test was found in 834 (16%) of the women and they were referred for colposcopy. VIA positivity was 11% whereas VILI positivity was 13%. Of the women referred for colposcopy, 13 were less than 20 years of age and 12 were over 60 years of age. The majority (809) were between 20-60 years.

Of the 834 women referred for colposcopy, 209 (25%) did not return for colposcopy. 625 women returned for the colposcopic evaluation and were tested for HIV, and 103 (16%) were HIV positive. See Table 2.

**Table 2:** VIA/VILI results, compliance with referral and HIV status

	Number	%
**VIA result**		
Positive	567	11.1
Negative	4 538	88.9

**Total**	**5 105**	**100**

**VILI result**		
Positive	664	13.0
Negative	4 441	87.0

**Total**	**5 105**	**100**

**Referral for colposcopy**		
Referred	834	16.3
Not referred	4 271	83.7

**Total**	**5 105**	**100**

**Compliance with referral**		
Returned for colposcopy	625	75.0
Did not return for colposcopy	209	25.0

**Total**	**834**	**100**

**HIV status (women who returned for colposcopy)**		
Positive	103	16.5
Negative	522	83.5

**Total**	**625**	**100**

		

Of the 625 colposcopied women 608 (97.3%) were between 20-60 years of age and were the ones included in the analysis of detection rates. One hundred and sixty nine (169) women had cervical lesions: 128, inflammation; 19, LGSIL; 13, HGSIL; 9, invasive cancer. Including colposcopic diagnosis of inflammation, the false positive screening by visual inspection alone, calculated as the proportion of women with a positive visual inspection but a negative colposcopy, was 72% (439 out of 608).

Detection rates of the different cervical lesions were higher among women aged 41-60 years compared to younger women aged 20-40 years (Table 3). Respectively, they were 55 per 1 000 and 20 per 1 000 for inflammation, 10 per 1 000 and 2 per 1 000 for LGSIL, 5 per 1 000 and 2 per 1 000 for HGSIL, 6 per 1 000 and 1 per 1 000 for invasive cervical cancer.

**Table 3:** Diagnosis based on positive colposcopy and detection rates for the different lesions by age group

Colposcopy diagnosis	Number screened N = 4119		Number screened N = 860		Total diagnosed	Detection rates per 1 000 women screened		
			
	Age: 20-40 years		Age: 41-60 years			Age (years)		
	**N**	**%**	**N**	**%**	**N**	**20-40**	**41-60**	**All ages**

Inflammation	81	63.3	47	36.7	128	19.7	54.7	25.7
LGSIL	10	52.6	9	47.4	19	2.4	10.5	3.8
HGSIL	9	69.2	4	30.8	13	2.2	4.7	2.6
Invasive cancer	4	44.4	5	55.6	9	1.0	5.8	1.8
Any lesion	104	61.5	65	38.5	169	25.2	75.6	33.9

Analysis is for women aged 20-60 years since only 2.5% of all women were outside that age range.

The 32 women with SIL (19 LGSIL and 13 HGSIL) underwent treatment by cryotherapy (31 women) or LEEP (1 woman). After three months of treatment a new colposcopy was carried out among the women who had been treated with cryotherapy: 1 woman had persistent LSIL and 1 had inflammation; both were HIV positive. Two women, both HIV negative, did not return after 3 months. All other 27 women had normal findings. There was no difference in the treatment outcomes of the HIV positive and HIV negative women after 3 months.

## Discussion

Our study found a high prevalence of HIV among the women referred for colposcopy after positive screening using visual inspection. Inflammation of the cervix uteri and low grade cervical lesions were significantly associated with HIV seropositivity. False positivity by visual inspection was high considering colposcopy diagnosis to be correct.

The study had some limitations. Biopsies were not taken when colposcopy results were considered normal or inflammation only, as we did not desire to add morbidity. Moreover, the study aimed to simulate conditions as they would be in a non-study situation of service provision. However, colposcopy tends to overestimate severity of lesions. We used only the colposcopy diagnosis in the analysis and thus might have overestimated the severity of the cervical lesions since colposcopy is not the gold standard of diagnosis [34].

The nurses were newly trained in screening using visual inspection. Though their skills improved over time, they had initial problems especially with accepting cervical ectopy as a normal finding. This contributed to the high false positivity at screening. Because of limitation of time and resources, the study ended before we could accumulate enough numbers to have enough power to emphatically study the effect of HIV seropositivity on treatment outcomes using cryotherapy.

Among women screened positive at visual inspection and referred for colposcopy, HIV prevalence at 16% was double that of the general Ugandan women population aged 15-49 years [14]. The higher HIV prevalence among women referred for colposcopy may imply that detectable cervical lesions using VIA/VILI are more prevalent among HIV positive women, or that a higher proportion of HIV positive women attend the postnatal and family planning clinics compared to the general population. Others may have heard of the screening service and came specifically for this. This may be true especially for those who knew their HIV status prior to the study. Women in Kampala who know their HIV status are usually more health conscious and are more likely to come for postnatal care and also to seek contraception [35].

Prevalence of inflammation was significantly higher among HIV positive women compared to HIV negative women. Of the 9 women with invasive cancer, 8 were HIV negative. Because of the very small numbers of lesions, we could not establish statistical associations between the different lesions and HIV serostatus or other explanatory variables. The findings of high prevalence of inflammation were expected, since other sexually transmitted infections causing cervical inflammation would lead to more false positive screening. The interaction between HIV and other sexually transmitted diseases of the reproductive health tract have been reported to be synergistic [36,37]. We didn't do biopsy lesions where the colposcopist diagnosed an inflammation (i.e. no precancerous lesion), therefore few lesions classified as inflammation may have been low grade precancerous lesions. Still the high HIV prevalence among the women referred for colposcopy would most likely imply a high prevalence of STDs and therefore cervicitis.

Cervical precancerous lesions are known to take years to progress. Luciani et al. [38] in a see and treat program in Peru found that cryotherapy was effective and cheap in treatment of precancerous lesions and could be used by low level health workers. They reviewed the patients after 1 year and found cure rates as high as 88% in all CIN and 70% for baseline diagnosis of CIN3. We had similarly good results after cryotherapy. Only 2 women (both HIV positive) had lesions on review after 3 months. 27 (20 HIV negative and 7 HIV positive) had no evidence of disease. However in view of the small numbers and the relatively short follow up time of 3 months, we could not make an emphatic conclusion about the effect of HIV serostatus on treatment outcome.

Considering colposcopy diagnosis as correct, we had very high false positive results of visual inspection through screening by the nurses. Out of the 608 women aged 20-60 years visually considered as positive, only 169 were colposcopically diagnosed with cervical lesions even though 128 of these were inflammation. Assuming that all the 169 women had dysplasia, it would mean that only 27.8% of the women should have been positive, which denotes a false positivity of the visual screening of at least 72%. The nurses were instructed to error on the positive side, so as minimize false negative screening as much as possible. Another possible reason for the high false positivity was the large number of women who had ectopy. This is quite confusing to the inexperienced nurses especially with use of Lugol's iodine when screening. Since 9 nurses were involved in our study, it was not possible for all of them to have attained the same level of skill. The findings of a high false positivity rate have implications for see and treat screening programs. Whereas some advocate treatment with cryotherapy after a positive VIA test ('see and treat'), others advocate for cryotherapy after VIA and positive colposcopy ('see-see and treat'). If we had adopted the first one, we would have over treated by 72% (439 out of 625). This has implications of side effects of treatment like cervical stenosis and sometimes incompetence, infections and persistent discharge. In this study where many women had inflammation, cryotherapy would have added to significant morbidity. Over treatment in 'see and treat' strategies has been noted to range between 1.2-83.3% for LGSIL and 12.3-83.3% for HGSIL. With use of colposcopy over treatment could be brought down to less than 30% overall [39]. There is evidence that nurses can be trained effectively as colposcopists [40-42]. It would be advisable to incorporate this colposcopy training in the 'see and treat' strategies for cervical cancer prevention. In the absence of colposcopists, it is imperative to ensure very intensive and continuous training for the nurses and close supervision so as to minimize over treatment.

Detection rates per 1 000 women screened were higher among the older women (41-60) years compared to younger women aged 20-40 years. This has implications for screening programs in low resource settings. With the few available resources, it would be wiser to target women at least above 30 years of age. A high loss to follow up of 25% means policy planners need to devise programs where the colposcopy and treatment is done the same day as the screening. Currently in Uganda cervical cancer prevention has at long last received the political attention long overdue. In addition to the above program in Mulago, there is an effort to roll out screening to other parts of the country, using visual inspection. In some of the programs, see and treat strategy is being used. Our findings will influence the need to invest in colposcopy training so as to adopt the see-see and treat strategy.

## List of abbreviations used

AIDS: acquired immunodeficiency syndrome; CD4: protein; CIN: cervical intra-epithelial neoplasia; HGSIL: high-grade squamous intraepithelial lesion; HIV: human immunodeficiency virus; HPV: human papillomavirus; LGSIL: low-grade squamous intraepithelial lesion; LEEP: the loop electrocautery excision procedure; RR: risk ratio; SIL: squamous intraepithelial lesion; STDs: sexually transmitted diseases; VIA: visual inspection with acetic acid; VILI: visual inspection with Lugol's iodine

## Competing interests

The authors declare that they have no competing interests.

## Authors' contributions

TM, FM and EW were involved in development of concept and full proposal, approval from ethical committees. TM provided for data collection entry and data cleaning. SS, TM and EW supported this project with data analysis. All authors participated in results interpretation, manuscript writing and text revision.
